# The transition to college: lived experiences of academically talented students with autism

**DOI:** 10.3389/fpsyt.2023.1125904

**Published:** 2023-07-05

**Authors:** Quinn Austermann, Nicholas W. Gelbar, Sally M. Reis, Joseph W. Madaus

**Affiliations:** Department of Educational Psychology, Neag School of Education, University of Connecticut, Storrs, CT, United States

**Keywords:** autism spectrum, high school & university, secondary transition to college, qualitative, autism

## Abstract

The experiences of autistic college students have become an increasing focus of research over the past 10 years. As a part of a larger research project, 40 successful autistic college students were interviewed about their experiences transitioning from high school to college. Participants reported being active participants in selecting colleges, but not receiving robust transition services during high school. They reported wanting additional opportunities in high school to develop executive function skills and to have more social opportunities. Further, they stressed the importance of developing greater independence while in high school.

## Introduction

In recent years, the number of students identified with autism spectrum disorder (ASD) has increased (1, 2), leading researchers to speculate that the number of college students with ASD will also increase (3–5). Academically talented or gifted students with ASD, often labeled as twice exceptional students with ASD (2eASD), are transitioning to higher education, yet little empirical research has focused on these students (6, 7). A recent systematic review of research on academically talented individuals with ASD found that little research is being conducted on this population (7). Of the 32 articles included in this review, 63% included data, yet no intervention studies had been published. While a small number of studies have focused on college transition for students with ASD [e.g., (4, 8, 9, 10)] none could be found that were specific to students who have ASD and academic talents and who matriculated at very competitive colleges. As young people who are 2eASD transition to college, it is important for secondary educators to understand their varied experiences to provide adequate support and opportunities to prepare for transition.

Giftedness, talent development, and ASD are all terms that represent complex exceptionalities. Definitions of giftedness and ASD continue to expand and adapt, but for the purpose of this article, 2eASD refers to students who are twice exceptional, meaning they possess both gifts/talents and one or more disabilities, in this case, ASD (11). Students who are 2eASD may exhibit difficulties with social interaction and an excessive focus on special interests (6, 11), which may cause teachers and neurotypical peers to view them as “quirky,” leading to a misidentification of giftedness and/or ASD.

Social and communication deficits are at the center of the core difficulties associated with ASD (10–12), yet a student’s services in their Individualized Education Program (IEP) no longer apply after high school graduation. Meaningful transition services should be individualized and based on thorough research and evidence-based practices. However, with limited research available about this population, many school professionals may be left feeling inadequately prepared to support students with ASD transition to college life. It is the student’s responsibility to disclose their disability to their college or university, yet communication deficits can affect students’ access to services. Elias and White (8) found reluctance from students when it came to disclosing ASD to their colleges, resulting in a lack of proper supports. This lack of support is often because they are not sought out, not because they are not available. Additionally, nongifted students with ASD tend to utilize educational services more often than students identified as academically gifted and with ASD (13).

## Transition experiences of 2eASD students

There is a need for current research on the experiences of 2eASD high school students, especially as more of these students are attending college (3, 5). Understanding the transition experiences of this unique population will help high school educators provide more meaningful transition planning and learning opportunities. Of the sparse research available, much is focused on parent perspectives, rather than the voices of students themselves. Four central themes are discussed that emerged from a review of the literature, including (1) the importance of inclusion, strength-based programs, and goal specific transition plans; (2) the need for effective strategies for educators to meet the needs of students with ASD; (3) the importance of peer relationships; and (4) the importance of balance in parent involvement. Each is described below.

### Inclusion, strength-based programs, and goal-specific transition plans

To best prepare students with 2eASD for success in college, their high school experiences must be designed in a way that matches students’ needs. While plans should be individualized, research suggests that students should be immersed in classes with neurotypical peers (14), and that programs should be designed to promote and develop strengths (11, 15, 16), and transition plans should be goal specific (14). As mentioned earlier, some research suggests that many college students with ASD are reluctant to disclose their disability (4, 8). Madaus et al. (17) conducted interviews with service providers at competitive post-secondary institutions and these findings suggest that some of the skills and traits that most affect the success of 2eASD students are related to social skills, self-advocacy, self-management, and motivation. Challenges related to these skills, combined with the reluctance to disclose their ASD, may lead to a challenging first-year experience at a competitive institution. Research suggests that some students in high school facilitate their own IEP meetings (18), which may help empower students when talking about their ASD and, in turn, enable them to feel more confident advocating for their needs upon entering college.

The research literature suggests that student experiences in high school should be engaging, strength-based, and, to an extent, resemble their future college experience, including being around neurotypical peers for much of the school day (11, 14, 15). These opportunities can enable 2eASD students to develop their communication and executive functioning skills, and also to explore their interests and talents.

Many students with ASD have specific interests and related strengths. Hillier et al. (10) interviewed parents for a study on prospective college students with ASD, reporting many student strengths that will help their transition to college. These included deep interests and intense knowledge in interest areas, and the parents note that these students have great potential to succeed with appropriate support (10). This study, along with others, recommends early college preparation and early arrival programs for students with ASD (11, 10, 16).

### Effective strategies for educators to meet the needs of students with ASD

The relationship between students and school staff is arguably one of the most important factors in an adolescent’s academic success (19). However, current research indicates that there is a lack of knowledge and training for educators when it comes to successfully teaching and understanding students with ASD (13, 20–24). Meeting the needs of this population of students is repeatedly a concern for staff, students, and parents. In a recent study of teachers, findings indicatated that students with ASD may be misunderstood as misbehaving because of a lack of teacher knowledge about ASD (21). This study also suggests that some students with ASD perform below grade level, which is because teachers do not know how to meet the needs of students with ASD, and also because students with ASD often do not seek help (21). The research indicates that teachers are not the only ones who recognize this barrier.

A gap seems to exist about educators’ knowledge about best practices and teaching strategies for students with ASD. In a study of high school students with ASD, the students reported that they believed their teachers had stereotypes surrounding ASD and lacked valid knowledge about students with ASD (20). When students are aware that their teachers have a lack of understanding in their disability, and therefore a lack of knowledge in how to support them, it can impede the student’s academic development. Furthermore, a qualitative study of parents of students with ASD lists parents’ top two areas of concern as “school personnel’s ability to meet students’ needs” and “appropriately trained teachers” (22). Therefore, in addition to creating strength-based and goal-specific plans for transition, it is essential that students with ASD receive appropriate instruction in the classroom to help prepare students with ASD for their transition to college.

### Peer relationships

Another critical factor influencing students’ success in school is their relationships with peers. For students with ASD, these relationships can either enhance or hinder their school experience. Students with ASD reported that their most positive experiences in high school are surrounding relationships they have formed and support they have received (20). Conversely, the downsides of high school are also related to social aspects, particularly bullying, loneliness, and feelings of being different (20, 24). This may lead to students with ASD isolating themselves; this lack of socialization also has negative effects on the reported emotions of these students (25). Teachers also report the importance of peer relationships for these students, noting that students with ASD tend to establish relationships with other students with ASD, which helps to minimize feelings of isolation (21). Furthermore, when K-12 schools and college universities closed their buildings during the COVID-19 pandemic, there were negative impacts on social relationships for some 2eASD students. In a study conducted by Madaus et al. (26), several 2eASD college students reported difficulties contacting professors, maintaining peer relationships, and connecting for group projects. Many students at the high school level with similar experiences may consider this another barrier to forming relationships with faculty and peers when they arrive at a college campus.

Opportunities for students with ASD to collaborate with other successful college students with ASD can be positive and empowering. Hillier et al. (10) developed a peer mentoring program to prepare high school students with ASD for college. They found that peer-mentoring was extremely effective, particularly when their mentor was an older peer with ASD who had successfully transitioned to college. In another study, students reported that it was important to them that their peers understand ASD and their needs (20). Programs like these can help students feel more comfortable around peers that are both neurotypical and autistic, resulting in increased confidence in social situations, and a sense of belonging in both the high school setting and during the transition to college.

### Importance of a balance in parent involvement

Parent perspectives are also highlighted in research conducted on high school students with ASD [e.g., (22, 27, 28)] suggesting that parents feel unprepared for their child’s postsecondary transition and that challenges exist about finding appropriate educational placements for their child (22, 28). One study about parents of students who are 2eASD found that parents suggest three areas of transition supports that should be emphasized: (1) enhancing independent living skills preparation, (2) post-secondary education-specific self-determination skill development, and (3) executive functioning and social skills support (27).

Parental involvement is critical, providing but so are opportunities for student independence in high school will also be integral in preparing for their transition. Examples of these opportunities from the literature include enabling students to facilitate their own IEP meetings (18), student participation in college immersion experiences during high school (11, 17) and allowing for high school students with ASD to develop clubs that foster their interests (25). This study includes valuable first-hand perspectives from students who are 2eASD about their experiences transitioning to competitive colleges and universities.

### Purpose of this study

Although historically there has been little focus on the transition experiences for 2eASD students who are 2eASD, Reis et al. (11, 15) have recently addressed this dearth of research. They conducted a qualitative study to identify successful strategies contributing to the academic success of 40 students identified as 2eASD who have been successful in highly competitive colleges. Semi-structured interviews were conducted with students who are 2eASD and had matriculated at competitive colleges and universities that yielded five specific strength-based teaching and instructional strategies students suggested contributed to their academic success. These high-school teaching and support strategies included: (1) dual identification as 2e, (2) interest-based extracurricular activities, (3) challenging and advanced classes, (4) use of strengths-based learning strategies, and (5) the establishment of strong and trusting relationships with teachers and counselors. While other researchers [e.g., (9, 10, 14, 21)] have addressed transition strategies for students with ASD, these findings are specific to students with academic talents and ASD who are successful at very competitive colleges.

The purpose of this qualitative study is to understand the lived experiences of successful college students who are academically talented and diagnosed with autism spectrum disorder. Specifically, we were interested in the postsecondary transition process for this population, and what participants identified as 2eASD found to constitute best practices, as well as challenges they encountered during the transition to their postsecondary institution. There is scant literature from the perspective of 2eASD students and we hope these findings contribute to meaningful transition planning practices for students with both academic talents and ASD.

## Methods

As part of a larger study (11), forty students with ASD who were enrolled in, or were recent graduates of, 4-year postsecondary institutions located in Western, Midwestern, and Northeastern states were interviewed regarding their experiences as a 2eASD student. Each participant attended a school rated as a highly competitive college or university in the *U.S. News and World Report 2021 Best National University Rankings* (11). The sample was primarily male (*n* = 27); fourteen participiants were freshman or sophomores and additional 14 were juniors or seniors. Four graduate students and eight students who were “between grades” comprised the sample. Please see the original study for a more comprehensive discussion of the study’s demographics.

While the methodological approach and findings from Reis et al. (11) helped to guide this study, the separate analysis conducted in this qualitative study focused on specific transition data not previously analyzed. The study included 40 semi-structured interviews, co-conducted by three researchers, which lasted between 45 and 90 min. A goal of the researchers was to make the participants feel welcome and comfortable in sharing their experiences. Prior to asking questions, each interviewer introduced themselves and discussed the background and aim of the study to build rapport with the participations. The protocol began by asking students open-ended questions about their interests and experiences that enabled students to talk about themselves in a way that did not alienate them (29). As the interview continued, each researcher asked content questions, but also probed for clarification, asked follow-up questions, and provided each participant with the opportunity to include any additional information. Questions related to transition included experiences related to living experiences, readiness for challenging content courses, ability to communicate with faculty and other adults, support from home and friends during transition, relationships with other students, study strategies used, projects completed, and other areas related to transition. Each participant was sent a formal thank you letter, given an incentive of a gift certificate, and confidentiality was reassured. After each interview was transcribed, both interviewers reviewed the transcript for accuracies and the transcript was also forwarded to the participant. Member checks allowed for participants to add or edit any information they felt necessary to reflect an accurate account.

Once all data were collected and transcribed, the participants were assigned pseudonyms, also used in this article, and data were deidentified. For the purpose of this study, questions about the college transition process were extracted and analyzed through a codebook thematic analysis (TA) approach. The TA approach to analyzing research is appropriate for interview data and offers a flexible approach to coding (30). The goal of TA is to derive themes from the data through interpretations, without restraint from structured methodologies or a set theoretical framework (31). Some qualitative scholars argue that data saturation is not important, but since this study is discovery oriented, the researchers agreed saturation was met after 40 interviews. The use of a codebook provided structure to an extensive data set (30). The researchers followed the six phases suggested by Terry et al. (32): familiarization, coding, theme development, reviewing themes, defining themes, and producing the report.

The transcripts were carefully read and reviewed by each researcher multiple times for familiarity. Due to an extensive amount of data, the transcripts were reorganized by question to allow for content coding. This study was particularly interested in the secondary transition experiences of the participants. After the researchers were familiar with the data set, independent initial coding began. Initial coding consisted of a combination of descriptive and *in vivo* coding (33). After the first round of coding, the researchers shared their codes and discovered overlap, repetitive codes, and patterns began to emerge. The researchers organized codes into eleven categories and created a codebook based on these categories. The data were then recoded based on these newly developed categories. These categories included: (1) integration/inclusive experiences; (2) strength-based interests; (3) time management; (4) peer relationships; (5) relationships with professors/teachers; (6) self-advocacy; (7) social skills; (8) activities of daily living; (9) difficulties with transition; (10) mental health challenges; and (11) study strategies. Researchers asked the participants their level of involvement in the transition planning process, about their parents/family’s help in transition to college, and if social and living skills were taught in high school. The participants’ responses were less detailed, and therefore quantified, for these questions.

After individually recoding the data with the new codebook, it was sent to the third researcher for interrater reliability. When the third researcher returned with feedback, the three researchers condensed the eleven categories into eight developed themes, as summarized in Table 1, that included: (1) time management; (2) learning/study strategies; (3) mental health and coping strategies; (4) academic preparation and engagement; (5) independent living skills; (6) navigating the college environment; (7) socialization; and (8) developing independence.

**Table 1:** Emergent themes from the data.

Theme	*n*	Description	Example
Academic preparation and engagement	65	Strength-based approaches, as well as negative experiences such as lack of support	“Academically, my high school got me ready. A lot of the classes were fast paced and also had high expectations. My high school did a good job. The content is different but I had the learning skills I needed.”
Navigating the college environment	52	Connecting to services (tutoring, disability services, mental health), differences between high school and college, and mentoring	“Um-tell them the truth about college. The truth is preferred. Tell them that it is hard, tell them about the grading systems, etc. I did not realize how different it was from high school.”
Time management	50	Creating a structure that allows students to authentically practice time management	“My issue was more about finding time and dealing with time management. I wasted a lot of time doing little things but not focusing on the big picture.”
Socialization	43	Positive (including using extra-curricular activities to foster socialization) and negative (including difficulties with roommates)	“Meeting new people was hard for me. I had met people from different groups but it was almost always one at a time or a meeting on-line, but this was especially challenging for me to meet lots of new people at one time.”
Mental health and coping strategies	32	Anxiety, finding support, taking a break, separation anxiety from home	“But also giving them the ability to destress, desensitize, and to just chill and recuperate and be themselves again. Again the social battery, everyone’s different, having that time to recharge is important”
Developing independence	31	Living alone, leaving home, and self-advocacy	“There was a lot of homesickness at first. Adapting to an independent lifestyle was difficult.”
Learning/study strategies	21	Including executive function skills, such as chunking assignments	“I had to … develop skills, and compensatory strategies for things like executive function.”
Independent living skills	15	Activities of daily living, including eating, hygiene, living with a roommate	“I think that that the most difficult part was taking care of stuff—maintenance, laundry, cleaning my room.”

The researchers, working together, found these eight themes to be representative of the codes and categories, and therefore, representative of the participants’ experiences of secondary transition. These themes were subsequently consolidated into three major findings. According to current or recently graduated college students at these very competitive universities, secondary transition for academically talented high school students with autism should include academic preparation, social and emotional skills and support, and development of independent living.

## Findings

Overall, participants indicated the desire to have increased opportunities to develop skills in all areas during high school. For example, participants were asked if they had been taught any independent living or social skills directly while in high school and 21 (53%) participants indicated that they were not taught any of these. Seven (18%) discussed having skills directly taught, and eight (20%) referred to narrow areas of skill being introduced or having a project in a course that involved some very specific skills (e.g., budgeting). For example, Penny stated: “We had a budgeting class, it was an elective about financial literacy and the economy, doing worksheets, it wasn’t its own structured program, just an elective. In terms of social skills, no, I had more when I was young.” In addition, when asked about their involvement in the transition to college, 32 (80%) participants indicated that they were highly involved. It is important to note that no participants specifically discussed the secondary transition process as indicated in IDEA, but they did suggest being involved in selecting the colleges to which they applied. As Avery explained: “I was heavily involved, it was mostly my decision, choosing my college and getting everything ready for going to college, things I needed. I had deciding power”.

Three findings emerged from these data that provide greater context to the overall findings. First, participants indicated the desire to have better developed academic and executive function skills before they entered college. Second, participants discussed more experiences involving social and emotional challenges than academic challenges during their transition to college. Finally, participants noted that developing greater independence after high school was a process that involved developing skills in multiple domains.

### Better academic/executive function skill development In high school

The first finding that emerged from data reflecting on participants’ academic preparation in high school related to their beliefs that their academic skills and executive function skills including time management and learning/study strategies should have been more fully developed before entering college. The majority of the participants (*n* = 32, 80%) noted that their high school had focused on the need for strong academic background skills to be successful in college. Direct quotes using pseudonyms for the participants are included to reflect their experiences while protecting confidentially. As Melissa stated: “academically, my high school got me ready. A lot of the classes were fast paced and also had high expectations. My high school did a good job. The content is different, but I had the learning skills I needed.” However, 16 of the participants (40%) indicated that they wished they had more opportunities to develop advanced academic skills in high school (sometimes generally and sometimes in areas of academic weakness). Austin’s comment is representative of participants’ perceptions:

I’d probably take more AP classes and get used to actually studying a lot and get used to difficult classes. I’d probably focus more on what major I want to do. I would have done more programming and found out more about what computer science is before choosing that major.

Matthew echoed this statement, adding “I would say that the toughest part of the transition was the physical pace of the classes—they were so fast! The content was difficult, and the pace was fast.”

In addition to desiring greater academic opportunities in high school, participants also indicated that they wished that they had opportunities to develop better time management (*n* = 27, 68%) and learning/study strategies (*n* = 14, 35%) in high school. Edgar, when asked about what the researchers should teach high school students with ASD, noted: “Strategies for using time well—teach them to not ever—never do things at the last minute. Chunk out your time so that you have enough time to do your work in time.” Gavin also suggested that “Students should start getting used to manage their time on their own but still have places they are expected to go to and things to get done and manage time well, practice that before college.” In terms of learning/study strategies, Adam indicated that he did not know how to study or take notes when he got to college and Georgia elaborated, explaining the need for: “Focusing tips. Being able to learn how to say no, put the computer down, and do the work. My self-control has been something I struggle with”.

### Social and emotional challenges

The second major finding that emerged from these data was that while academic transition to college can be challenging for this group, social and emotional challenges are also very important to identify and overcome. Twenty-two (55%) participants reported the importance of learning from negative social experiences, for as Sasha noted: “The social stuff was hard for me, though—being around people like me helped at PROGRAM but then I got to COLLEGE, and I needed to know how to deal with neurotypical people.” Jackson refined this, explaining:

Social issues—going in, I told myself that I would make more friends. But that has not happened. But I have to change how I act. I have no friends. I only have friends from high school and elementary school. There is only one kid that I talk to occasionally.

In particular, Evan reported: “Social issues were very hard. I was not used to socializing with people I do not know. Slightly better now but not what it should be.”

In order to develop socially during the transition to college, several participants discussed the importance of joining clubs and suggested that high school students should be encouraged to get involved in extracurricular activities. Jackson stated: “Socially, potentially, for kids like me, they should have made me socialize more.” Kobe reinforced this finding, explaining, “It was easy to transition socially as soon I joined the gaming club, I was able to have friends.” When asked to provide specific suggestions, Susan suggested: “So, prepare them for alcohol issues and dating and other social challenges that you will be exposed to—such as certain types of friendships.”

Participants (*n* = 19, 48%) also discussed the importance of learning to independently cope with mental health challenges. Matthew noted that more information about coping with mental health challenges needs to be provided to students, and Ashley concurred, stating: “there were just a lot of emotions and a lot of change that was hard for me to adjust to, and I did not have the right resources or people to help me with that.” Georgia suggested “also giving them the ability to destress, desensitize, and to just chill and recuperate and be themselves again. Again, the social battery, everyone’s different, having that time to recharge is important.” Eva discussed the need to understand changes in order to be successful:

Changes in routine have been difficult. Right now, I’m relaxed because it’s halfway through the semester so I’m in the groove. It’s hard when there’s something new. Sometimes when classes are canceled my days are weird because I need the normal time. Sometimes it’s hard to take care of myself because my body doesn’t tell me what’s going on, like with food.

### Developing independence

Participants believed in the importance process to develop the skills to independently navigate the college environment including using independent living skills, navigating the differences in the college environment versus high school, and that there is a transition phase in which you have to learn to be responsible for yourself. Twenty-nine (73%) of the participants specifically discussed the importance of high school students understanding the differences between the high school and college environments. Edgar noted: “I think that the most difficult part was that in high school, I had a high volume of work but in college, I had to focus less on volume and much more on advanced quality of work.” Austin added: “Emphasize that in college you’d have to study more, and it’ll be more academically rigorous. I wish my advisor had pointed that out more and also helped with deciding a major.” Mary refined this by stating: “The expectations were different and often I would encounter professors who were really nonspecific in their language and that can be hard when someone dances around what they mean.” Susan gave the following advice for us assisting with this part of the transition to college:

You should include things that help students understand resources that are available to them. Over-expose them to services that will help them in the long run. There should not be threshold for services. Let people know what is available to help them. Also, a basic review of challenges with transitions—there are so many. Some of the ways that transitions happen are not clear.

Twenty participants (50%) reported that initially living away from home and being independent was a challenge. Sasha explained, “A lot of the challenges was living away from my parents and living alone initially.” Jacob concurred: “It was hard living away from my mom because she’s the rock in my life.” Ten participants also reported that they wished that their independent living skills had been better developed when they were in high school, for as Rachel explained:

I wish they had had some sort of home ec, something similar. For the most part at home, I was taught things you’d be taught in a home ec class, but it was compartmentalized into this is what I do at home instead of in school.

## Discussion and implications

The findings of this study suggest that while the participants were very involved in selecting the colleges they subsequently attended, they were not always ready to succeed in those environments. Few reported that they participated in or receive targeted instruction or exposure to experiences focused on developing independent living or social skills and relations in high school. As a group, our participants had more developed self-determination skills than in previous studies, potentially because they are academically talented or gifted (8, 13, 17). However, the absence of systematic instruction in some academic and social skills highlights a research-to-practice gap as multiple studies have discussed the importance in developing these skills for individuals with ASD (17, 20, 24). It is important to note that all of the participants in this study were involved and engaged in inclusive classrooms and school communities, considered essential for the social and academic development of high school students with ASD (10, 14, 21), but most still wished that they had more direct opportunities to learn the independent living and social relationship skills that they found they lacked in college.

The participants experienced success highly competitive colleges in the United States, but slightly less than half also indicated that regretted not having learned and being explicitly taught more intensive academic and study skills while they were in high school to help to prepare them for college. This finding mirrors the results of surveys that have been conducted with college accessibility service providers, who often report that some college students with ASD are academically underprepared and need to develop better executive function and study skills in order to thrive in the college environment (17, 34). An easier college immersion experience, either during the summer in a residential program or a virtual class might have enabled these students to better understand and meet some of the challenges college life before arriving for their freshman year. The research literature in the field suggests that early college experiences, combined with goal setting and individualized plans, may help ease the transition for college-bound students with ASD (11, 10, 15).

Similar to previous studies (26, 27), our sample reported experiencing significant social and emotional challenges during the transition to college. This reflects the literature findings [e.g., (17, 20, 24, 26)] regarding the importance of providing social supports and opportunities for students with autism in high school. Many of our participants indicated that high school students with ASD should be encouraged to become involved in extracurricular activities so their social world and experiences can be broadened before college and so they can reflect and learn from any negative social situations that they encounter.

Improving peer interaction would also make a difference as strong peer relationships can help to strengthen social and emotional learning and background experiences for college students with ASD (11, 15). To help address concerns over peer relationships among students with ASD, some research suggests additional opportunities for inclusive enriching activities (25, 28) and our findings also demonstrate that providing opportunities for extra-curricular experiences, clubs, and interest-based activities with peers is helpful to social and emotional growth. Our work supports other research (10, 25) that suggests the importance of activities and clubs, including movie trivia, ultimate frisbee, and video game clubs that promote an increase in engagement with neurotypical peers (25). Increasing opportunities for students to integrate and bond over similar interests create more positive socialization experiences. Additional support for pursuing interests also emerged in participant responses in the Reis et al. (11, 15) studies, where 90% of students reported involvement in extracurricular activities, many of whom attributed this involvement to their success in college.

The two themes of increasing academic and social skills prior to college coalesce in a third theme in which participants discussed the importance of developing independence. This finding aligns with the concept of developing self-determination skills, which has extensive empirical support [e.g., (4, 25)]. High school staff should encourage opportunities for high school students with ASD to develop greater independence. This can be accomplished in multiple ways, as supports can be given during early years and then gradually reduced over the course their high school career, as students develop necessary skills. Students can be encouraged to learn how and when to request their accommodations from their general education teachers, which is a skill they have to utilize in college. They should also be encouraged to take challenging classes, which push them to learn the learning and study strategies they will need when they enter college. As parent perspectives are valuable, it is important to understand the proves of becoming more independent from parents as students consider college opportunities. When students enter college, parents contact with professors and counselors ends, and these are often relationships that parents indicated would be hard for them to relinquish (4). The students in this study recognize the importance of parent support in transition and the emotional support they offer, but also reported looking forward to the independence and freedom that accompanies college life. A careful balance must be established between the students’ desire for independence and their reluctance to leave familiar surroundings and support (4). Finally, as noted previously, college bound students with ASD should be encouraged to become involved in extracurricular activities so they can develop advanced social skills and learn to work in groups.

## Limitations

Several limitations exist in this study. While the larger themes and findings are derived from the individual experiences of the participants, it is important to note that these findings may not be generalizable to all individuals who are 2eASD. Qualitative design was used to address the aims of this study, as we felt it best reflected the voice and perspective of the students in our sample, however, including mixed-methods may have made this study more robust. Additionally, voluntary participation may suggest our sample is more motivated and have greater social and self-determination skills than the larger population of 2eASD individuals. The data are self-reported, and therefore may include inaccuracies and social desirability may have been reflected in the responses. However, we are confident that our sample is representative of the target population, as they have been accepted to very competitive colleges and universities and received services for their autism diagnosis. Furthermore, although we focused on recruiting a diverse population, the majority of our participants were male and white. A quarter of our participants identified as women and 16% were culturally diverse.

## Conclusion

Many students diagnosed with ASD have unique talents and strengths, making them excellent candidates for highly competitive colleges and universities. Little research exists that specifically focuses on the transition experiences of this population from high school to college. The findings from this study suggest that 2eASD students have great potential for college success, yet specific work needs to be accomplished to prepare these students during their high school years. Particularly, employing goals and learning opportunities that enhance executive functioning, social and emotional skills, and independent living will help 2eASD students transfer these skills in the college environment. For example, high school staff should explore opportunities within and outside of the curriculum in high school to enable these students to better develop these skills before students attend college. Teachers should also be encouraged to work with families to provide suggestions for developing these skills prior to matriculation in college. Our findings suggest that students did not receive explicit instruction on how to develop study skills. Teaching these types of study skills is recommended for students who are college bound, especially at highly competitive colleges. We suggest continued research on the transition experiences of students who are 2eASD and further research on the high school experiences that contributed to college success for these students.

## Data availability statement

The raw data supporting the conclusions of this article will be made available by the authors, without undue reservation.

## Ethics statement

The studies involving human participants were reviewed and approved by UConn-Storrs Institutional Review Board. The patients/participants provided their written informed consent to participate in this study.

## Author contributions

NG, SR, and JM developed the questionnaire and conducted the interviews. QA and NG conducted the data analysis for this study. All authors contributed to the article and approved the submitted version.

## Funding

This research was supported by a grant from the Jacob K. Javits Gifted and Talented Students Education Program.

## Conflict of interest

The authors declare that the research was conducted in the absence of any commercial or financial relationships that could be construed as a potential conflict of interest.

## Publisher’s note

All claims expressed in this article are solely those of the authors and do not necessarily represent those of their affiliated organizations, or those of the publisher, the editors and the reviewers. Any product that may be evaluated in this article, or claim that may be made by its manufacturer, is not guaranteed or endorsed by the publisher.
